# SIRT6 Depletion Sensitizes Human Hepatoma Cells to Chemotherapeutics by Downregulating MDR1 Expression

**DOI:** 10.3389/fphar.2018.00194

**Published:** 2018-03-06

**Authors:** Yang Q. Xia, Ren J. Hua, Chen Juan, Zhou H. Zhong, Cheng S. Tao, Ren Fang, He Lin, Gong Rui, Chen Yong

**Affiliations:** ^1^Department of Hepatobiliary Surgery, First Affiliated Hospital, Chongqing Medical University, Chongqing, China; ^2^The Key Laboratory of Molecular Biology of Infectious Diseases, Chinese Ministry of Education, Chongqing Medical University, Chongqing, China

**Keywords:** hepatocellular carcinoma, SIRT6, chemotherapeutic agents, chemosensitivity, MDR1, C/EBPβ

## Abstract

Multidrug resistance (MDR) due to overexpression of MDR1 is a major obstacle that hinders the treatment of hepatocellular carcinoma (HCC). In this study, we explored the function and underlying molecular mechanism of SIRT6 in MDR of HCC. Chemotherapeutic agents (doxorubicin, cisplatin, and sorafenib) treatment increased SIRT6 mRNA and protein level in two HCC cell lines in a dose-dependent manner. SIRT6 depletion resulted in decreased cell viability and increased apoptosis in HCC cells treated with chemotherapeutic agents. Mechanistically, SIRT6 depletion reduced MDR1 transcription by targeting its promoter in HCC cells treated with chemotherapeutic agents. Consistently, the protein level of MDR1 was also reduced in SIRT6-depleted HCC cells. Further studies indicated that SIRT6 depletion may suppress CCAAT/enhancer binding protein β (C/EBPβ), to act as a transcriptional activator of MDR1 in HCC cells treated with chemotherapeutic agents. Importantly, forced expression of MDR1 could attenuate the apoptosis induced by chemotherapeutic agents in SIRT6-depleted cells. Taken together, these results indicated SIRT6 depletion enhanced chemosensitivity of human hepatoma cells by downregulating MDR1 expression through suppressing C/EBPβ. SIRT6 may serve as a novel target to enhance chemosensitivity in HCC cells.

## Introduction

Hepatocellular carcinoma (HCC) is one of the most common and third-leading causes of cancer deaths worldwide (El-Serag and Rudolph, 2007). Approximately, 0.7 million of people die each year from the liver cancer (Park et al., 2008). Surgical resection is not applicable for the majority of patients who undergo metastasis or tumor recurrence. Therefore, comprehensive chemotherapy has become one of the effective treatments for the patients mentioned above. For example, doxorubicin and cisplatin which are cytotoxic drugs used in systemic therapy achieve low objective response rates (typically < 10%). A tyrosine kinase inhibitor sorafenib considered as a breakthrough can extend median survival by little more than a year (Stotz et al., 2015). Especially, sorafenib has become standard therapy for advanced-stage HCC in recent years due to its good tolerance (Llovet et al., 2008; Benson et al., 2009; Cheng et al., 2009; Lencioni et al., 2014; Stotz et al., 2015). Unfortunately, multidrug resistance (MDR) limits the efficacy of chemotherapy drug in HCC patients. Numerous studies have shown MDR is an important reason why HCC patients have low sensitivity to diversified drugs (Petraccia et al., 2003). Some mechanisms of MDR which have been reported at present are listed as follows: (1) increasing drug efflux by membrane transporters (Chun et al., 2015), such as MDR1 protein, MDR-related protein; (2) inhibiting apoptotic signaling pathway by Survivin gene (Or et al., 2014). Therefore, it’s urgent to explore the mechanism of MDR and improve the susceptibility of HCC cells to chemotherapeutics.

Silent information regulator 6 (SIRT6) is an NAD^+^-dependent deacetylase which belongs to the sirtuin (SIRT) family. In HCC, there were some evidences showing that SIRT6 promoted tumor growth. Our group demonstrated that SIRT6 inhibited HCC cells apoptosis via the Bax-dependent signaling pathway *in vitro* and *in vivo* (Ran et al., 2016). Namgyu and colleagues found that SIRT6 depletion promoted cellular senescence by increasing DNA damage in HCC (Lee et al., 2016). It also has reported that SIRT6 was closely associated with MDR in various cancers. SIRT6 depletion increased chemotherapeutic agents sensitization in prostate cancer (Liu et al., 2013), breast cancer (Khongkow et al., 2013), and non-small cell lung cancer (Azuma et al., 2015). Also, Sociali’ group showed that SIRT6 inhibitors Quinazolinedione could enhance cancer cells susceptibility to chemotherapeutic agents (Sociali et al., 2015). As far as we know, there is no report describing the relationship between SIRT6 and chemosensitivity in HCC.

Our group previously reported that SIRT6 is high-expression in a subset of HCC tissues and cells. SIRT6 induced apoptosis of HCC cells by regulating Bax-dependent apoptotic pathway (Ran et al., 2016). In this study, we found that chemotherapeutic agent treatment increased SIRT6 mRNA and protein level in two HCC cell lines. SIRT6 depletion could sensitize HCC cells to chemotherapeutic agents by inhibiting MDR1 expression. Our data suggested a role for SIRT6 in chemosensitivity of HCC cells and identified it as a novel therapeutic target.

## Materials and Methods

### Cell Lines and Reagents

The HCC cell line SK-Hep-1 was gained from the American Type Culture Collection and Huh-7 cells were obtained from the Health Science Research Resource Bank. The Dulbecco’s modified Eagle medium (DMEM) was purchased from the Corning Incorporated. The fetal bovine serum (FBS) was purchased from the Gibco BRL. All cell lines were seeded in DMEM with 10% FBS. Chemotherapeutic agents including doxorubicin (Sigma, D1515), cisplatin (Sigma, P4394), and sorafenib (Selleckchem, S7397) were dissolved in DMSO at a concentration of 1.5, 1, and 4.5 mg/mL, respectively. They were added to the culture media to make final concentrations indicated in section “Results.” The following antibodies and plasmids were acquired from listed companies: anti-SIRT1 (CST, #9475), anti-SIRT2 (CST, #12650), anti-SIRT3 (CST, #2627), anti-SIRT4 (Santa, sc-135798), anti-SIRT5 (CST, #8782), Anti-SIRT6 (Novus, NB100-2522), anti-SIRT7 (Sigma, S5947), Anti-MDR1 (Wanleibio, WL02395), Anti-C/EBPβ (Wanleibio, WL01710), Anti-acetyl-Histone H3 (Ac-Lys9) (Millipore, 07-352), Anti-GAPDH (Santa, sc-365062), SIRT6 expression vector (OriGene, RC202833), MDR1 expression vector (Addgene, #10957), C/EBPβ expression vector (Vigene Biosciences). Non-targeting shRNA or SIRT6 short hairpin RNA was obtained from Shanghai Genechem Company Limited. The sequences of shCont are 5′-GCAACAAGATGAAGAGCACCAA-3′ and the sequences of shSIRT6-1 and shSIRT6-2 are 5′-GCTACGTTGACGAGGTCATGA-3′ and 5′-GCCTCTGACTTGCTGTGTTGT-3′, respectively.

### Western Blotting Analysis

The protein level was detected by Western blotting analysis as described in our previous work (Song et al., 2016), the brief process is described as follows. SK-Hep-1 and Huh7 cells were treated with various chemotherapy drugs and cultivated for 48 h after depletion or forced expression of SIRT6. Then the cells were lysed using RIPA lysis buffer with protein inhibitor and total proteins were obtained by centrifuging to remove sedimentation. BCA Protein Assay (Thermo) was used to measure the protein concentrations. Proteins with equal quantity (30 μg) were separated by sodium dodecyl sulfate-polyacrylamide gel electrophoresis (SDS-PAGE) and transferred onto nitrocellulose membrane (GE Healthcare). Membranes were incubated with primary antibodies overnight and then with secondary antibodies linked to horseradish peroxidase. The protein bands were detected using immobilon western chemiluminescent HRP substrate (Millipore).

### Real-Time qPCR

Total RNA was extracted using TRNzol Reagent (Invitrogen) and cDNA was acquired by an iScript^TM^ cDNA Synthesis Kit (Bio-Rad, Richmond, CA, United States). Relative gene mRNA expression levels were quantified by FastStart Universal SYBR Green Master Mix (Roche Diagnostics). β-Actin was used as an internal control. The expression values of target genes represent the means ± SD of three independent experiments. Values were analyzed using the 2^-ΔΔCt^ method. The primer sequences were designed by our group and were listed in Supplementary Table 1.

### MTS Assay

SK-Hep-1 and Huh7 cells were treated with various chemotherapy drugs and cultivated for 48 h after depletion or forced expression of SIRT6. To detect cell viability as a reaction to chemotherapy drugs after depletion or forced expression of SIRT6, CellTiter 96^®^ AQ_ueous_ One Solution Cell Proliferation Assay (Promega) was used according to the manufacturer’s instruction.

### Apoptosis Assay

The HCC cells were treated with various chemotherapy drugs and cultivated for 48 h after depletion or forced expression of SIRT6. Cells were firstly stained using Annexin V-FITC Apoptosis Detection Kit (Beyotime Biotechnology) according to the manufacturer’s instruction. Then, the percentage of apoptosis cells was tested by Flow Cytometry (BD Accuri C6) as described earlier (Tao et al., 2016).

### Luciferase Reporter Assay

MDR1 promoter fragment including the region of -1587 ∼ +336 was subcloned into the pGL3-basic vector. The promoter and pRL-TK used to normalize the transfection efficiency were cotransfected with shCont or shSIRT6. Luciferase activity was determined by the Dual-luciferase Reporter Assay System (Promega) in accordance with the manufacturer’s instruction.

### Chromatin Immunoprecipitation Assay (ChIP)

Cells with different treatments were cross-linked in 1% formaldehyde at room temperature for 10 min and the DNA was sheered to a length between 200 and 1000 bp by sonication. The supernatants were incubated with the antibody specific and protein A/G magnetic beads at 4°C overnight. Subsequently, the DNA was retrieved from Protein/DNA complexes at 62°C for 2 h and purified. A region of the MDR1 promoter was amplified from the DNA samples by qRT-PCR using the sense primer F 5′-CTTCCTCCACCCAAACTTATCCT-3′ and the antisense primer R 5′-ATTCACAGGCAGTTTGGACAAGA-3′.

### Co-immunoprecipitation Assay

The protein magnetic beads (Millipore) were prepared according to the manufacturer’s protocol. 4 μg SIRT6 antibody (Novus, NB100-2522) and 500 μg protein were mixed in a rotator shaker at 4°C overnight. Then the binding reaction was added to the washed protein magnetic beads for 2 h at 4°C. Beads were washed for four times in wash buffer and were detected using Western blotting analysis. We have described as previously reported (Ran et al., 2016).

### Statistical Analysis

Data gap analysis between the groups was measured by Student’s *t*-test or one-way ANOVA. Correlations between SIRT6 and MDR1 were evaluated using Spearman’s σ rank test. Data were represented as means ± SD of three independent experiments at least. The value of *P* < 0.05 was considered statistically significant. All statistical analyses were carried out by using SPSS 19.0 software (IBM Corporation).

## Results

### SIRT6 Expression Was Upregulated Under the Treatment of Chemotherapeutics in Human Hepatoma Cells

**FIGURE 1:**
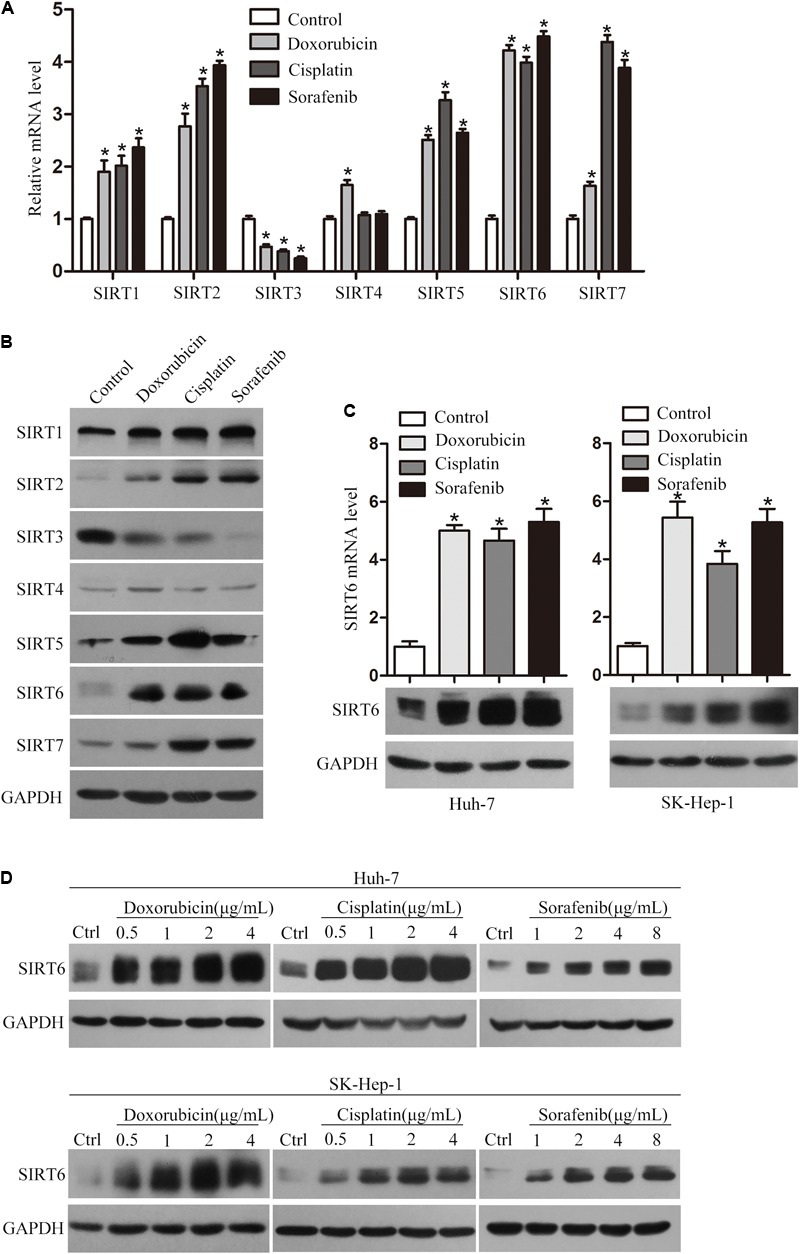
Chemotherapeutics promoted SIRT6 expression in HCC cells. The mRNA **(A)** and protein **(B)** levels of SIRT1 to SIRT7 were examined in Huh-7 cells treated with chemotherapeutic agents (doxorubicin, cisplatin, and sorafenib) for 48 h by using qPCR and Western blotting analysis. β-Actin was used as an internal control for qPCR. GAPDH protein expression was used as a loading control for Western blotting analysis. ^∗^*P* < 0.05 vs. control. **(C)** The mRNA and protein expression levels of SIRT6 were examined by using qPCR and Western blotting analysis. Huh-7 and SK-Hep-1 cells were exposed to the doxorubicin (1 μg/mL), cisplatin (2 μg/mL), and sorafenib (4 μg/mL) for 48 h, respectively. The mRNA expression level of β-actin was used as an internal control for qPCR analysis and GAPDH protein expression was used as an internal control for Western blotting analysis. ^∗^*P* < 0.05 vs. control. **(D)** The protein expression level of SIRT6 was tested in Huh-7 and SK-Hep-1 cells treated with various concentrations of the three chemotherapeutics for 48 h by using Western blotting analysis.

To determine whether chemotherapeutic agents could alter sirtuin family members expression, Huh-7 cells were treated with doxorubicin, cisplatin, and sorafenib, respectively. Sirtuin family members expression were detected by qPCR and Western blotting analysis. As shown in **Figures 1A,B**, chemotherapeutic agent treatment increased the mRNA and protein expression of SIRT1, SIRT2, SIRT5, SIRT6, and SIRT7. In contrast, SIRT3 expression decreased, which was reported by our group previously (Tao et al., 2016). In particular, the most significant increase has been observed in SIRT6 which is further studied in this study (**Figures 1A,B**). Simultaneously, treatment of chemotherapeutic agents in Huh-7 and SK-Hep-1 cells resulted in increased expression of SIRT6 mRNA and protein levels (**Figure 1C**).

To determine whether the expression of SIRT6 in HCC cells was associated with drug concentration, Huh-7 and SK-Hep-1 cells were treated with a series concentrations of chemotherapeutics, respectively. The results showed chemotherapeutic agents treatment enhanced SIRT6 expression in a dose-dependent manner (**Figure 1D**). These data suggested that SIRT6 may play a role in the drug resistance of HCC cells.

### SIRT6 Depletion Enhanced the Chemosensitivity of HCC Cells to Chemotherapeutic Agents

**FIGURE 2:**
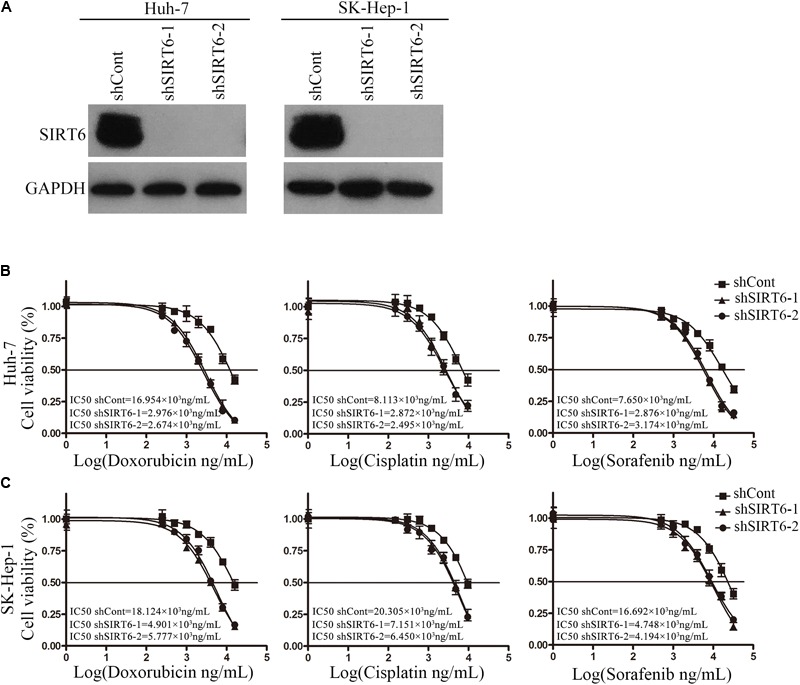
SIRT6 depletion reduced chemotherapeutic agents-induced cell viability in HCC cells. **(A)** The depleted efficacy of SIRT6 was tested by Western blotting analysis in Huh-7 and SK-Hep-1 cells. GAPDH was used as an internal control. Huh-7 **(B)** and SK-Hep-1 **(C)** cells depleted SIRT6 were exposed to various concentrations of doxorubicin, cisplatin, and sorafenib for 48 h, respectively. The cell viability was detected by using MTS assay.

**FIGURE 3:**
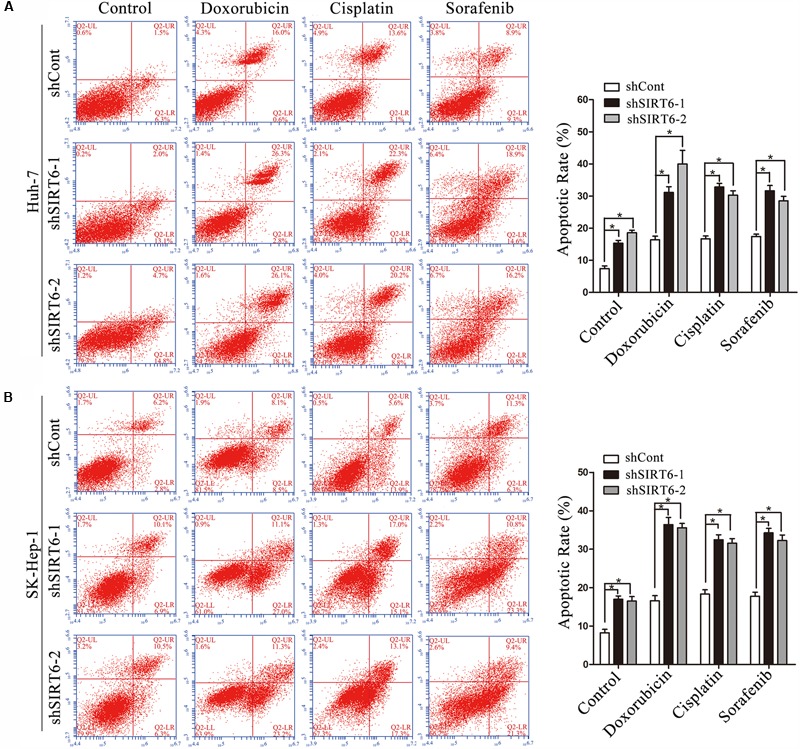
SIRT6 depletion increased the sensitivity of HCC cells to chemotherapeutics. **(A,B)** The cells depleted SIRT6 were exposed to doxorubicin (1 μg/mL), cisplatin (2 μg/mL), and sorafenib (4 μg/mL) for 48 h, respectively. The apoptosis was analyzed by flow cytometer with Annexin V/PI in Huh-7 **(A)** and SK-Hep-1 **(B)** cells. ^∗^*P* < 0.05 vs. shCont.

To investigate the function of SIRT6 in drug resistance of HCC, we first depleted the SIRT6 expression in HCC cell lines. The depleted efficiency of SIRT6 in two HCC cell lines was confirmed by Western blotting analysis (**Figure 2A**). SIRT6 depletion markedly increased the cellular susceptibility of Huh-7 and SK-Hep-1 cells to chemotherapeutics which were evidenced by using MTS assay (**Figures 2B,C**). The effect of SIRT6 depletion on chemosensitivity of HCC was further examined by flow cytometry. Chemotherapeutic agent treatment significantly increased the apoptotic rate of Huh-7 and SK-Hep-1 cells. Moreover, SIRT6 depletion further increased the apoptotic rate of chemotherapeutic agent-treated cells (**Figures 3A,B**). These results indicated SIRT6 depletion could improve drug sensitivity of HCC cells to chemotherapeutics.

### SIRT6 Depletion Increased Chemosensitivity via Downregulating MDR1

**FIGURE 4:**
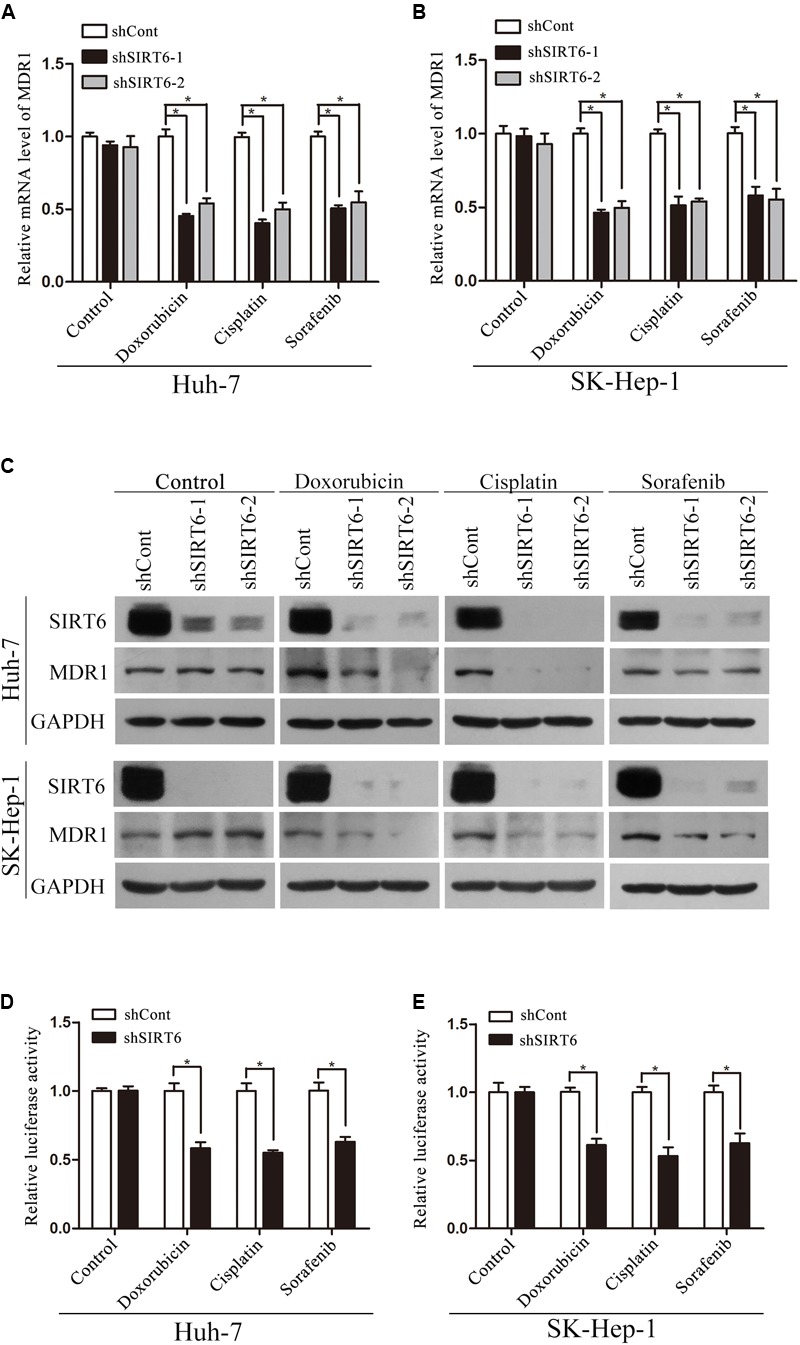
MDR1 expression was downregulated in SIRT6-depleted cells under the treatment of chemotherapeutics. The mRNA expression level of MDR1 was determined in Huh7 **(A)** and SK-Hep-1 **(B)** cells under the treatment of chemotherapeutics (doxorubicin, cisplatin, and sorafenib) by using qPCR analysis. β-Actin was used as an internal control. ^∗^*P* < 0.05 vs. shCont. **(C)** MDR1 expression was measured in SIRT6-depleted Huh7 and SK-Hep-1 cells under the treatment of chemotherapeutics by Western blotting analysis. GAPDH was used as an internal control. **(D,E)** The promoter activity of MDR1 gene was detected by using a dual-luciferase reporter assay. Huh7 **(D)** and SK-Hep-1 **(E)** cells depleted SIRT6 were transfected with reporter constructs containing the MDR1 promoter fragment under the treatment of chemotherapeutics. ^∗^*P* < 0.05 vs. shCont.

**FIGURE 5:**
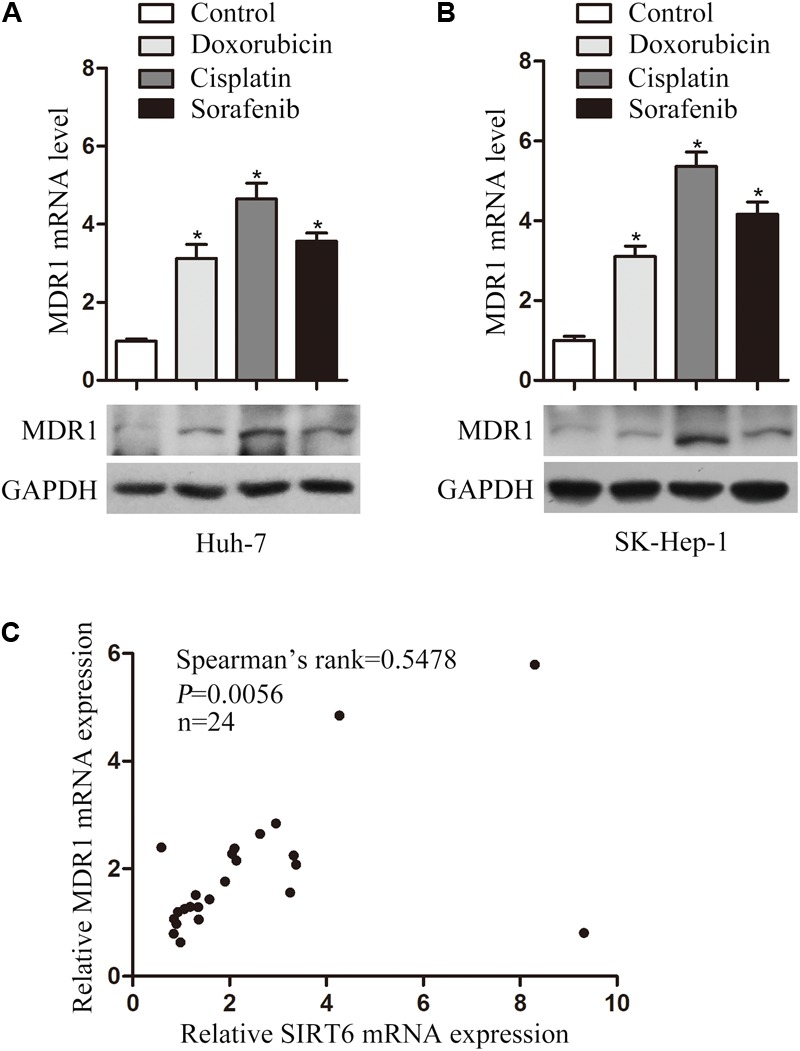
Chemotherapeutic agents increased MDR1 expression in HCC cells. The mRNA and protein levels of MDR1 were examined in Huh-7 **(A)** and SK-Hep-1 **(B)** cells treated with chemotherapeutic agents (doxorubicin, cisplatin, and sorafenib) for 48 h by using qPCR and Western blotting analysis. β-Actin was used as an internal control for qPCR. GAPDH protein expression was used as a loading control for Western blotting analysis. ^∗^*P* < 0.05 vs. control. **(C)** SIRT6 and MDR1 mRNA level were tested in 24 paired HCC tissues by using qPCR. Correlative analysis revealed a positive correlation between MDR1 mRNA and SIRT6 mRNA by Spearman’s σ rank test. β-Actin was used as an internal control for qPCR. *P* = 0.0056.

To further explore the mechanism underlying SIRT6 regulated multidrug-resistance, we screened six MDR-related genes, including MDR1, SOD, GSTP1, MRP, LRP, and TOP2B and several apoptotic cell death-related genes by using qPCR in SIRT6-depleted cells. The results showed the mRNA level of MDR1 was markedly downregulated in SIRT6-depleted cells under the treatment of chemotherapeutics (**Figures 4A,B**). However, SIRT6 had no significant effect on the other genes expression including SOD, GSTP1, MRP, LRP, TOP2B (Supplementary Figures 1A–E) and BCL2, BCL2L1, BCL2L2, BCL2L11, BCL2L12, P21, P27 (Supplementary Figures 2A–G). Western blotting assay showed that SIRT6 depletion had no effect on the MDR1 gene in HCC cells without the treatment of chemotherapeutic agents. However, SIRT6 depletion significantly reduced the expression of MDR1 in HCC cells with the treatment of chemotherapeutic agents (**Figure 4C**). Moreover, we tested the mRNA and protein levels of MDR1 in the two HCC cells treated with chemotherapeutic agents. Both qPCR and Western blotting analysis showed that chemotherapeutic agents significantly increased MDR1 expression (**Figures 5A,B**). These data indicated that MDR1 expression in liver cancer cells was induced under chemotherapeutic agent to facilitate cell survival. Importantly, SIRT6 depletion may inhibit MDR1 expression at transcriptional level to increase the sensitivity of the liver cancer cells to chemotherapeutic agents.

To confirm this hypothesis, we cloned MDR1 promoter region into a pGL3-Basic plasmid. Significantly, MDR1 promoter activity was reduced in the SIRT6-depleted HCC cells under the treatment of chemotherapeutics (**Figures 4D,E**). These results suggested SIRT6 depletion promoted cell chemosensitivity by suppressing MDR1 gene promoter activity. Additionally, we analyzed the correlation of SIRT6 and MDR1 expression in 24 paired HCC tissues by using qPCR. There was a positive correlation between SIRT6 mRNA and MDR1 mRNA levels (Spearman’s rank = 0.5478, *P* = 0.0056) (**Figure 5C**). The data indicated that SIRT6 might regulate MDR1 expression *in vivo*.

### Forced Expression of SIRT6 Decreased Chemosensitivity by Upregulating MDR1

**FIGURE 6:**
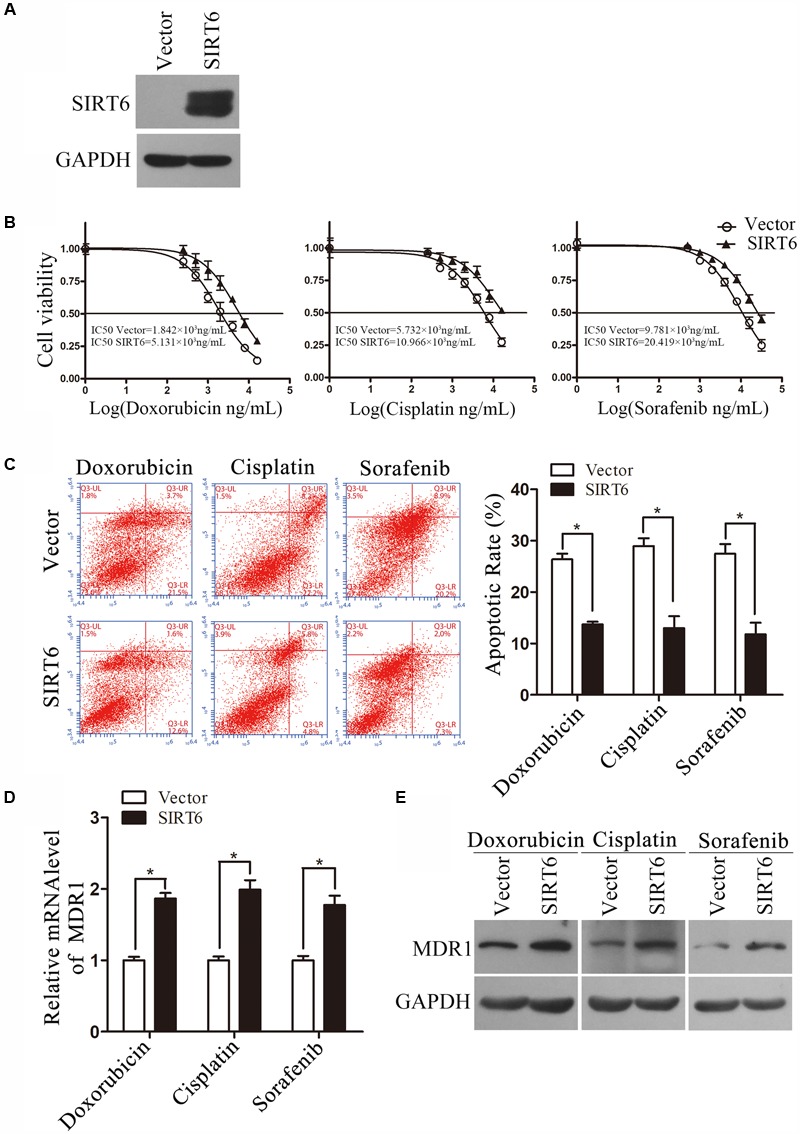
Forced expression of SIRT6 enhanced chemoresistance in HCC cells. **(A)** The forced expression efficacy was detected by Western blotting analysis. GAPDH protein expression was used as a loading control for Western blotting analysis. **(B)** The cells overexpressing SIRT6 were exposed to various concentrations of doxorubicin, cisplatin, and sorafenib for 48 h, respectively. The cell viability was tested by using MTS assay. **(C)** The cells forced expression of SIRT6 was exposed to doxorubicin (1 μg/mL), cisplatin (2 μg/mL), and sorafenib (4 μg/mL) for 48 h, respectively. The apoptosis was analyzed by flow cytometer with Annexin V/PI in SK-Hep-1. ^∗^*P* < 0.05 vs. Vector. **(D,E)** The mRNA and protein level was measured by using qPCR and Western blotting analysis. ^∗^*P* < 0.05 vs. Vector. β-Actin was used as an internal control for qPCR. GAPDH protein expression was used as a loading control for Western blotting analysis.

To further examine the functional role of SIRT6 in drug resistance of HCC, SK-Hep-1 cells overexpressing SIRT6 was exposed to doxorubicin, cisplatin, and sorafenib, respectively. The forced expression efficiency of SIRT6 in SK-Hep-1 cells was confirmed by Western blotting analysis (**Figure 6A**). The results showed forced expression of SIRT6 increased chemoresistance in SK-Hep-1 cells (**Figure 6B**). Furthermore, flow cytometry results showed a decreased apoptotic rate of SK-Hep-1 cells overexpressing SIRT6 under the treatment of chemotherapeutic agents (**Figure 6C**). Mechanistic study found that both the mRNA and protein level of MDR1 in SK-Hep-1 cells exposed to chemotherapeutic agents were increased by forced expression of SIRT6 (**Figures 6D,E**). These data indicated forced expression of SIRT6 enhanced chemoresistance by upregulating MDR1 expression.

### MDR1 Is Downregulated by C/EBPβ in SIRT6-Depleted Cell

**FIGURE 7:**
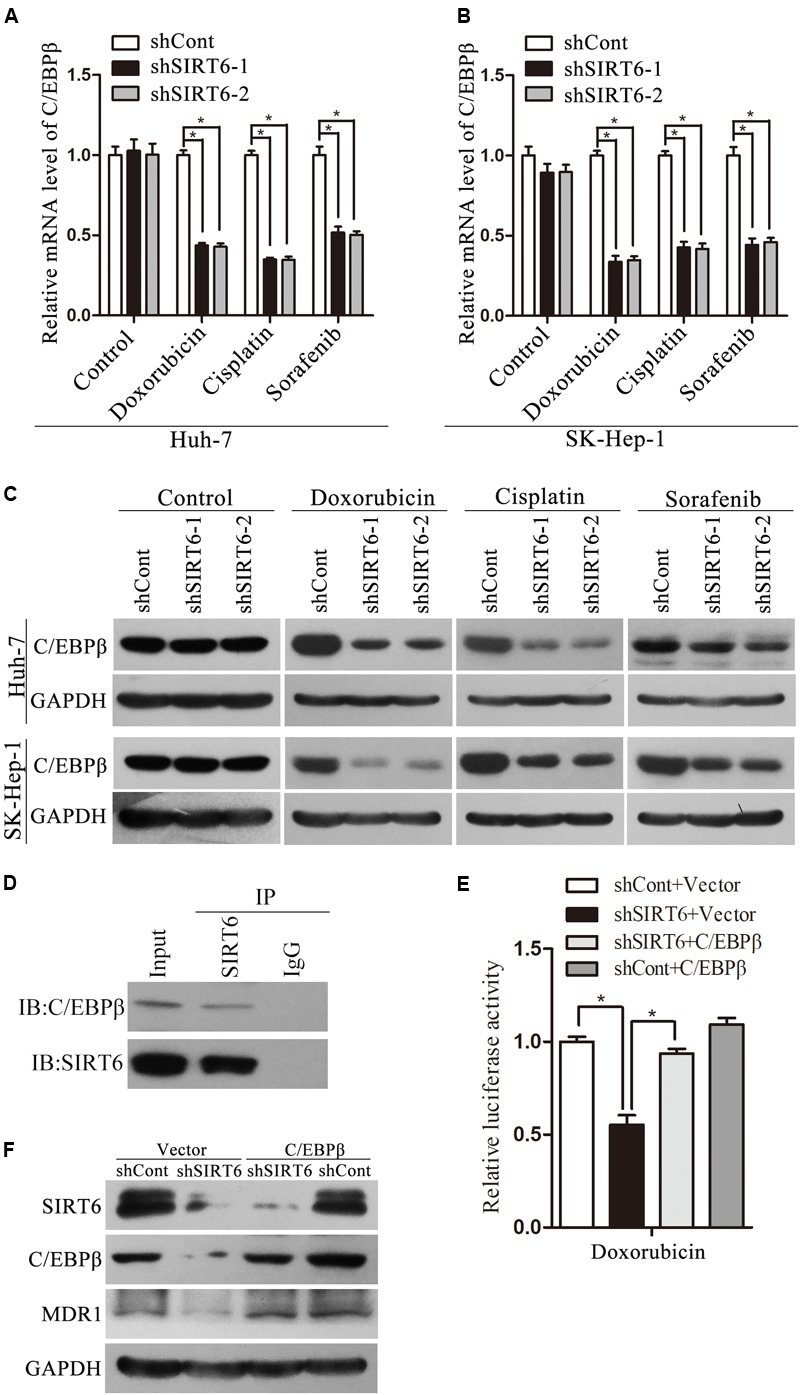
SIRT6 depletion inhibited MDR1 expression by downregulating C/EBPβ expression in HCC cells. The mRNA expression level of C/EBPβ was tested in Huh7 **(A)** and SK-Hep-1 **(B)** under the treatment of chemotherapeutics (doxorubicin, cisplatin, and sorafenib) by using qPCR analysis. β-Actin was used as an internal control. ^∗^*P* < 0.05 vs. shCont. **(C)** C/EBPβ protein expression was measured in SIRT6-depleted Huh7 and SK-Hep-1 cells treated with chemotherapeutics by Western blotting analysis. GAPDH was used as an internal control. **(D)** The association relationship between SIRT6 and C/EBPβ was detected by co-immunoprecipitation assay in Huh-7 cells under the treatment of doxorubicin (1 μg/mL). **(E)** C/EBPβ and shSIRT6 plasmids were cotransfected into Huh7 cells with the treatment of doxorubicin (1 μg/mL). The promoter activity of MDR1 gene was detected by using a dual-luciferase reporter assay. ^∗^*P* < 0.05 vs. shSIRT6+Vector. **(F)** C/EBPβ and shSIRT6 plasmids were cotransfected into Huh7 cells with the treatment of doxorubicin (1 μg/mL). The protein level of MDR1 was measured by Western blotting analysis.

We have previously found that SIRT6 was recruited to promoter of Bax promoter where it deacetylated histone H3K9 and thereby inhibited Bax transcription (Ran et al., 2016). However, in our study, the acetylation of H3K9 in MDR1 promoter was not changed in response to SIRT6 depletion (Supplementary Figure 3A). This data suggested that the acetylation of H3K9 on MDR promoter region was not regulated by SIRT6. Therefore, we further investigated whether SIRT6 increased MDR1 transcription by targeting certain transcription factor. Both GENECARDS and PROMO database predicted the transcription binding sites of several transcription factors including C/EBPβ, c-Jun, P53 and SP1, which were found in MDR promoter region. To demonstrate whether SIRT6 mediates these transcription factors in the chemosensitivity of HCC cells, we screened C/EBPβ, c-Jun, P53, and SP1 by using qPCR in SIRT6-depleted cells exposed to chemotherapeutic agents (Supplementary Figures 3B–D). The mRNA level of C/EBPβ was remarkably decreased in SIRT6-depleted cells under the treatment of chemotherapeutic agents (**Figures 7A,B**). However, SIRT6 depletion had no significant effect on the mRNA level of c-Jun and P53 in HCC cells treated with chemotherapeutic agents (Supplementary Figures 3C,D). The mRNA levels of SP1 were reduced in SIRT6-depleted cells treated with or without chemotherapeutic agents (Supplementary Figure 3E). Next, the protein level of C/EBPβ was detected by Western blotting analysis. Consistently, depletion of SIRT6 decreased C/EBPβ protein level in Huh-7 and SK-Hep-1 cells treated with chemotherapeutic agents (**Figure 7C**). Moreover, co-immunoprecipitation assay was applied to determine whether SIRT6 associated with C/EBPβ. Our data showed that there was an interaction between SIRT6 and C/EBPβ in Huh-7 cells exposed to chemotherapeutic agents (**Figure 7D**). These data indicated that SIRT6 depletion downregulated C/EBPβ in HCC cells treated with chemotherapeutic agents.

It has reported that C/EBPβ activated the human MDR1 gene by interacting with the MDR1 promoter via the region within -128 to -75 in human Cancer Cells (Chen et al., 2004). To evaluate the effect of C/EBPβ on the induction of MDR1 expression, we performed reporter gene assay and Western blotting analysis in Huh-7 cells exposed to doxorubicin. Importantly, ectopic expression of C/EBPβ reversed the suppressive effect of SIRT6 depletion on the promoter activity and protein level of MDR1 (**Figures 7E,F**). Taken together, these data suggested that SIRT6 depletion enhanced the chemosensitivity by repressing C/EBPβ expression, subsequently induced inhibition of MDR1 gene promoter activity in HCC cells.

### Reversal MDR1 Recovered Multidrug Resistance in HCC Cells

**FIGURE 8:**
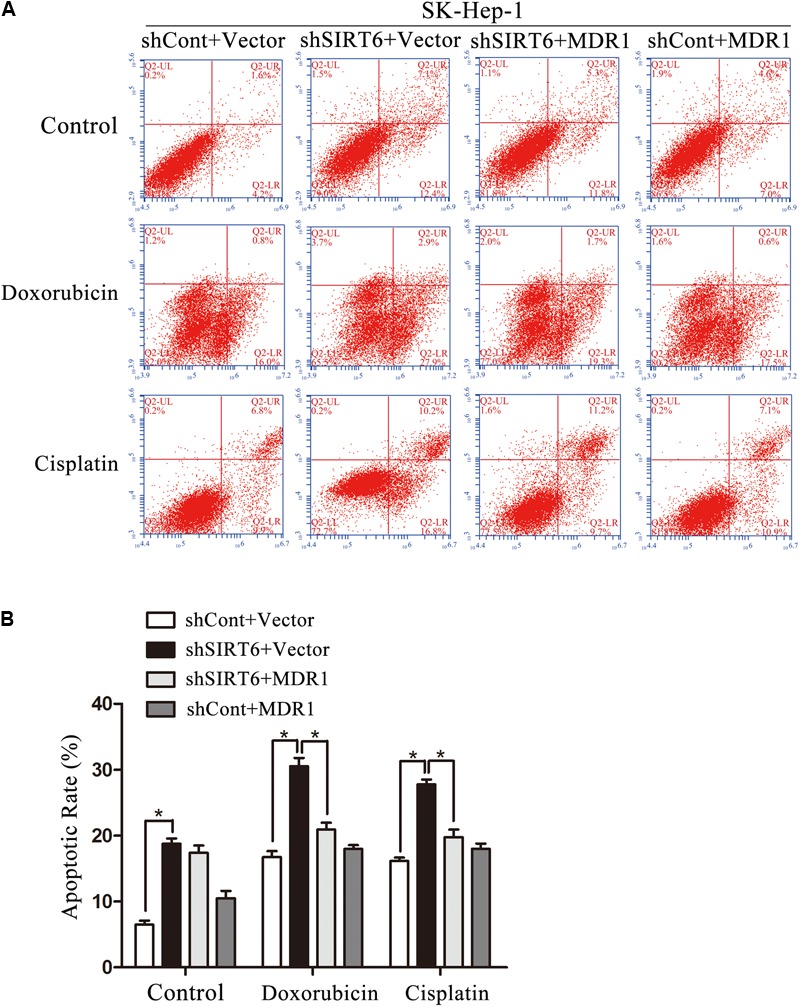
The forced expression of MDR1 recovered the multidrug resistance (MDR) of HCC cells. **(A,B)** MDR1 and shSIRT6 plasmids were cotransfected into SK-Hep-1 cells with the treatment of doxorubicin (1 μg/mL), cisplatin (2 μg/mL). The apoptosis was analyzed by flow cytometer with Annexin V/PI in SK-Hep-1 **(A,B)**. ^∗^*P* < 0.05 vs. shSIRT6+Vector.

We next determined whether forced expression of MDR1 could reverse increased drug sensitivity in HCC cells. MDR1 and shSIRT6 plasmids were cotransfected into SK-Hep-1 cells with the treatment of chemotherapeutic. Intriguingly, forced expression of MDR1 dramatically decreased cell apoptosis induced by SIRT6 depletion (**Figures 8A,B**). These results showed that SIRT6 depletion could sensitize HCC cells to chemotherapy by inhibiting MDR1 expression.

## Discussion

Multidrug resistance is one of the major drawbacks in liver cancer chemotherapy, which is herein related to cancer recurrence and therapeutic failure (Petraccia et al., 2003; Hu and Zhang, 2012). Therefore, the enhancement of chemosensitivity and determination of its mechanism in HCC may facilitate the HCC patient treatment in the future. It has been exhibited in our previous work that SIRT6 was upregulated in HCC cells and tissues. SIRT6 depletion suppressed cell proliferation, whereas forced expression of SIRT6 promoted cell growth in HCC (Ran et al., 2016). However, the function of SIRT6 in HCC chemosensitivity is not clear. In this study, we firstly observed that SIRT6 mRNA and protein level were upregulated in HCC cells treated with chemotherapeutic agents including doxorubicin, cisplatin, and sorafenib. Compared with other family members, SIRT6 had the most significant increase in HCC cells following exposure to chemotherapeutic agents. This alteration may be a cytoprotective response to chemotherapeutic agents and indicated that SIRT6 may play an essential role in drug sensitivity of HCC. Functional studies showed that SIRT6 depletion resulted in decreased cell viability and increased apoptosis in HCC cells treated with chemotherapeutic agents. Meanwhile, forced expression of SIRT6 facilitated cell survival in response to chemotherapeutic agents. The biological role of SIRT6 in HCC chemosensitivity is consistent with previous studies that SIRT6 depletion or inhibitor enhanced chemosensitivity (Liu et al., 2013; Sociali et al., 2015) in prostate cancer and breast cancer, respectively. Taken together, these data suggested SIRT6 depletion may play a sensitizing effect in HCC chemotherapy. Hence, we deduce that SIRT6 depletion combining with chemotherapeutic agents could be possibly provided as a novel treatment strategy in HCC. However, present studies are preliminary, and the sensitizing role of SIRT6 inhibitor should be further validated in HCC cells and animal model.

The mechanism of SIRT6 depletion mediating chemosensitization was further investigated in this study. We have screened six MDR-related genes in SIRT6-depleted cells with the treatment of chemotherapeutics by using qPCR. Results showed that only MDR1 mRNA level was significantly reduced under chemotherapeutic treatment in SIRT6-depleted cells. Next, Western blotting analysis demonstrated SIRT6 depletion also downregulated MDR1 protein level in HCC cells treated with chemotherapeutic agents. Furthermore, we constructed pGL3-Basic containing the MDR1 promoter to examine the effect of SIRT6 on MDR1 transcription. The data showed that SIRT6 depletion reduced MDR1 transcription by targeting its promoter in HCC cells treated with chemotherapeutic agents. Importantly, forced expression of MDR1 abolished SIRT6 depletion-induced apoptosis in HCC cells exposed to chemotherapeutic agents. Previous studies revealed that MDR1 plays a crucial role in chemosensitivity of HCC. Liu’ group showed that blocking of JNK signaling pathway enhances HCC cells sensitivity to cisplatin by reducing MDR1 expression (Liu et al., 2016). In addition, Wu et al. (2016) reported that the effect of MDR in HCC was reversed by metformin through suppressing NF-κB signaling to downregulate MDR1 expression. All the studies mentioned above support a fact that SIRT6 functions on liver cell chemosensitivity, at least in part, by manipulating MDR1 expression *in vitro*. Moreover, the analysis by using qPCR revealed that the correlation between SIRT6 and MDR1 in 24 paired HCC tissues is positive. The data further suggested that SIRT6 may regulate MDR1 expression *in vivo*.

Subsequently, we found that SIRT6 induced the expression of C/EBPβ in HCC cells treated with chemotherapeutic agents by using the databases and qPCR analysis. C/EBPβ is a critical transcription factor belonging to C/EBPs family, which can bind to DNA enhancer region. The relationship between SIRT6 and C/EBPβ is not clear yet. Previous reports indicated that SIRT1 was upregulated by NAMPT and NAD+ levels, subsequently bound and activated C/EBPβ in myeloid cells (Skokowa et al., 2009). In this study, we assumed that SIRT6 may interact with C/EBPβ. Co-immunoprecipitation assay demonstrated that SIRT6 indeed interplay with C/EBPβ in HCC cells exposed to chemotherapeutic agents. It is also known that C/EBPβ promotes MDR1 transcription by interacting with the MDR1 promoter in human cancer cells (Chen et al., 2004). We supposed that C/EBPβ may regulate MDR1 expression by activating MDR1 promoter. This hypothesis was validated by the results that forced expression of C/EBPβ rescued SIRT6’s effect on the promoter activity and protein level of MDR1.

In summary, SIRT6 depletion increased HCC cell sensitivity to chemotherapeutics via downregulating transcription factor C/EBPβ and subsequently inhibiting MDR1 expression. It may provide a novel therapeutic strategy by targeting SIRT6 to enhance chemosensitivity of HCC cells.

## Author Contributions

CY conducted the experimental design. YX and CY drafted the manuscript. YX and RH performed most of the experiments and analyzed the experimental data. RH and ZZ organized and helped to perform MTS and apoptosis assay. YX, RH, ZZ, CT, RF, HL, and GR contributed to conduct western blotting analysis and luciferase reporter assay. CY and CJ helped to compute and analyzed the experimental data. All authors contributed the interpretation of the data, revised the manuscript critically, and approved the final manuscript.

## Conflict of Interest Statement

The authors declare that the research was conducted in the absence of any commercial or financial relationships that could be construed as a potential conflict of interest.
