# Supplementary material for: SIRT6 Depletion Sensitizes Human Hepatoma Cells to Chemotherapeutics by Downregulating MDR1 Expression
Source: Front Pharmacol. 2018 Mar 6;9:194. doi: 10.3389/fphar.2018.00194 (PMC5845756; doi:10.3389/fphar.2018.00194)
Supplement: Supplementary file 1 [file Table_1.DOCX]

**Table S1 Primers of qPCR**

| Primer names | Sequences |
| --- | --- |
| SIRT6 sense | 5’GCAGTCTTCCAGTGTGGTGT3’ |
| SIRT6 anti-sense | 5’CCATGGTCCAGACTCCGT3’ |
| MDR1 sense | 5’CAGCTGTTGTCTTTGGTGCC3’ |
| MDR1 anti-sense | 5’TGGCAATGCGTTGTTTCTGG3’ |
| SOD sense | 5’ACAAAGATGGTGTGGCCGAT3’ |
| SOD anti-sense | 5’AACGACTTCCAGCGTTTCCT3’ |
| GSTP1 sense | 5’CCAAGTTCCAGGACGGAGAC3’ |
| GSTP1 anti-sense | 5’TGCCATTGATGGGGAGGTTC3’ |
| MRP sense | 5’GAAGCAGCCGGTGAAGGTTG3’ |
| MRP anti-sense | 5’CCTCCACCTCCTCATTCGCA3’ |
| LRP sense | 5’TGGCTGGTAACAGTGCAGGA3’ |
| LRP anti-sense | 5’CAGGGTGGTGATGGGCACAA3’ |
| TOP2B sense | 5’AAACCTGGCCAGCGGAAAGT3’ |
| TOP2B anti-sense | 5’ACTTCCCACAAAGTTCTGAGCCA3’ |
| BCL2 sense | 5’TTGAGGAAGTGAACATTTCGGTG3’ |
| BCL2 anti-sense | 5’AGGTTCTGCGGACTTAGGTC3’ |
| BCL2L1 sense | 5’AGGCAGGCGACGAGTT3’ |
| BCL2L1 anti-sense | 5’TTCCCATAGAGTTCCACAAA3’ |
| BCL2L2 sense | 5’TAAGCTGAGGAGAAGGGTT3’ |
| BCL2L2 anti-sense | 5’CGCCAGATCAGAGAAGGTG3’ |
| BCL2L11 sense | 5’GGCAAAGCAACCTTCTGATG3’ |
| BCL2L11 anti-sense | 5’CTTGTGGCTCTGTCTGTAGG3’ |
| BCL2L12 sense | 5’GGTCCAAGAGCAGCTGAAA3’ |
| BCL2L12 anti-sense | 5’AGGCCAGCTTCTGGTTAATG3’ |
| p21 sense | 5’CGACTGTGATGCGCTAATG3’ |
| p21 anti-sense | 5’TCTCGGTGACAAAGTCGAAG3’ |
| p27 sense | 5’AAACGTGCGAGTGTCTAACG3’ |
| p27 anti-sense | 5’CTCTGCAGTGCTTCTCCAAG3’ |
| C/EBPβ sense | 5’ACTACGAGGCGGACTGCTTG3’ |
| C/EBPβ anti-sense | 5’GAGGAAGTCGTGGTGCTGCC3’ |
| c-Jun sense | 5’AAGGAAGCTGGAGAGAATCG3’ |
| c-Jun anti-sense | 5’TTAAGCTGTGCCACCTGTTC3’ |
| p53 sense | 5’AACAACACCAGCTCCTCTCC3’ |
| p53 anti-sense | 5’CTCATTCAGCTCTCGGAACA3’ |
|  |  |
|  |  |
|  |  |
|  |  |
|  |  |
|  |  |
|  |  |
|  |  |
|  |  |
|  |  |
|  |  |
|  |  |
