# Supplementary material for: SIRT6 Depletion Sensitizes Human Hepatoma Cells to Chemotherapeutics by Downregulating MDR1 Expression
Source: Front Pharmacol. 2018 Mar 6;9:194. doi: 10.3389/fphar.2018.00194 (PMC5845756; doi:10.3389/fphar.2018.00194)
Supplement: Supplementary file 2 [file Presentation_1.PDF]

Supplementary Figure legends

Supplementary Fig.1 Multidrug resistance-related genes had no significant change in SIRT6-depleted cells. (A-F) The mRNA level of SOD (A), GSTP1 (B), MRP (C), LRP (D), and TOP2B (E) were tested by using qPCR analysis in SIRT6-depleted cells with the treatment of chemotherapeutic agents. β-actin was used as an internal control for qPCR.

Supplementary Fig.2 Apoptotic cell death-related genes had no significant change in SIRT6-depleted cells. (A-F) The mRNA level of BCL2 (A), BCL2L1 (B), BCL2L2 (C), BCL2L11 (D), BCL2L12 (E), P21 (F) and P27 (G) were tested by using qPCR analysis in SIRT6-depleted cells with the treatment of chemotherapeutic agents. β-actin was used as an internal control for qPCR.

Supplementary Fig.3 C/EBPβ, c-Jun, P53 and SP1 were screened by qPCR analysis. (A) ChIP assay with anti-H3K9Ac was performed in Huh-7 cells depleted SIRT6 under the treatment of doxorubicin. H3K9 acetylation at the MDR1 promoter (mean ± SD) is shown relative to input. (B-E) The mRNA level of C/EBPβ (B), c-Jun (C), P53 (D) and SP1 (E) were detected in Huh-7 cells depleted SIRT6 treated with doxorubicin for 48h by using qPCR. β-actin was used as an internal control for qPCR. *P<0.05 vs. shCont.
