# Supplementary figures and images for: SIRT6 Depletion Sensitizes Human Hepatoma Cells to Chemotherapeutics by Downregulating MDR1 Expression
Source: Front Pharmacol. 2018 Mar 6;9:194. doi: 10.3389/fphar.2018.00194 (PMC5845756; doi:10.3389/fphar.2018.00194)

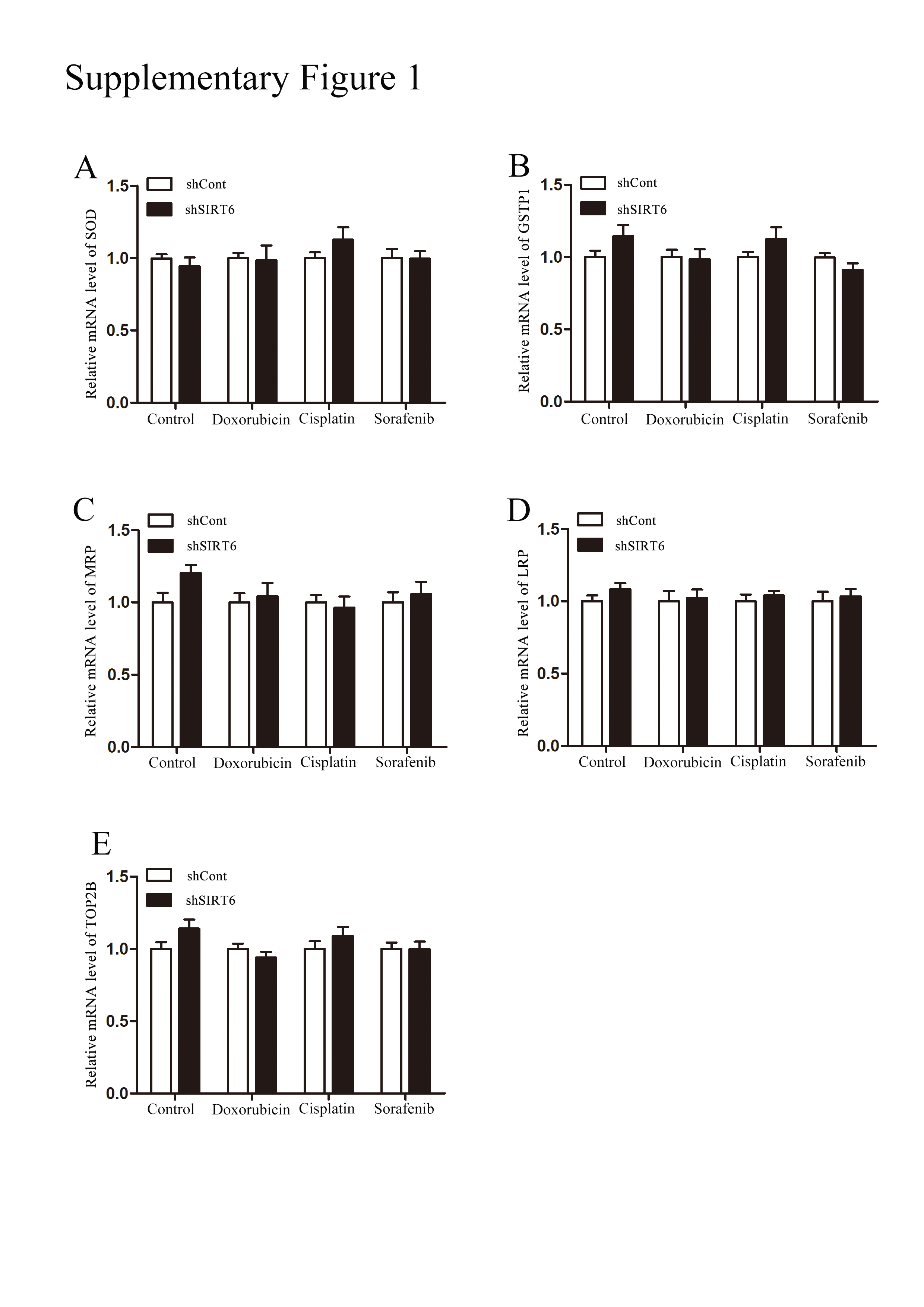

Supplement: Supplementary file 3 [file Image_1.TIF]

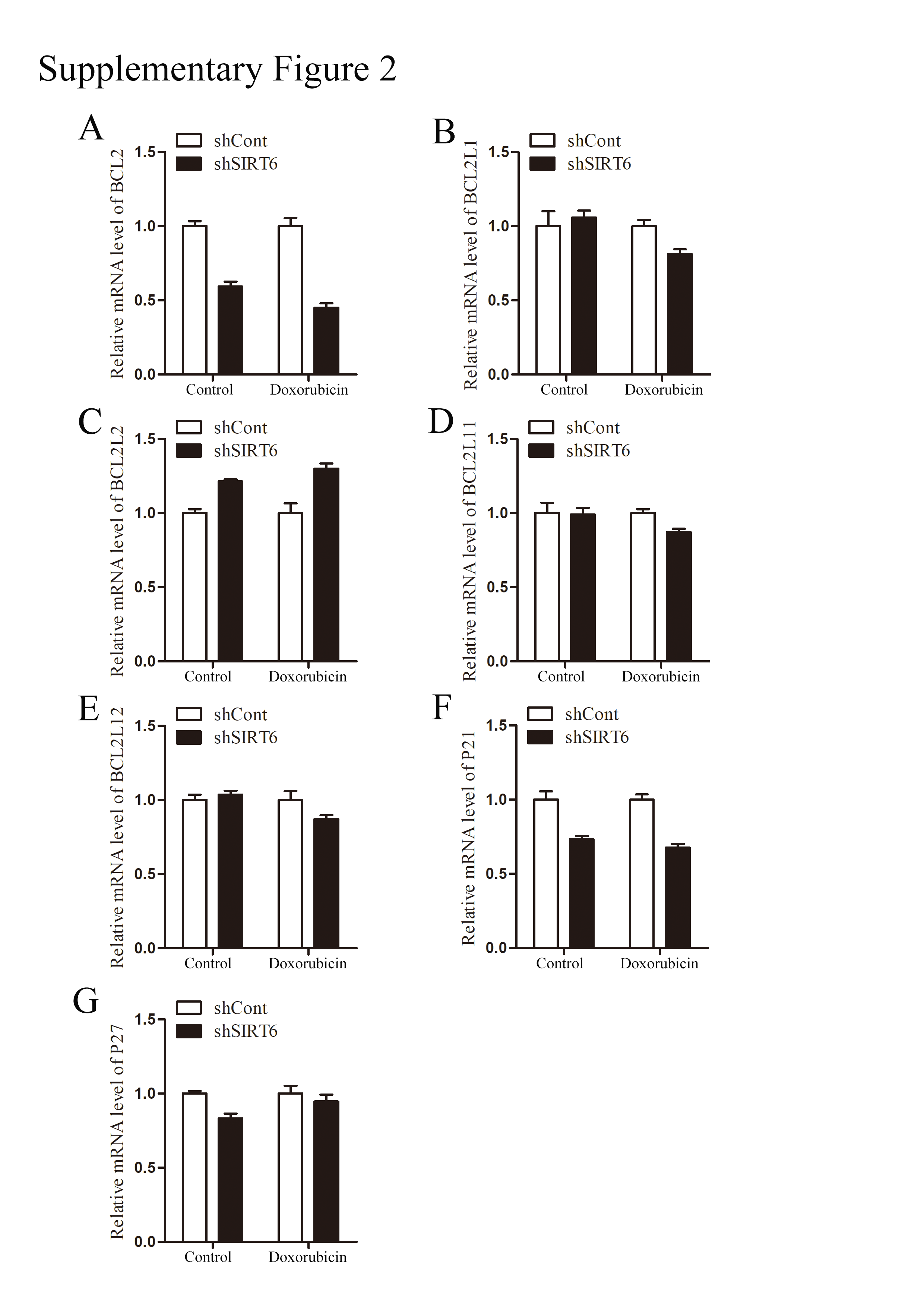

Supplement: Supplementary file 4 [file Image_2.TIF]

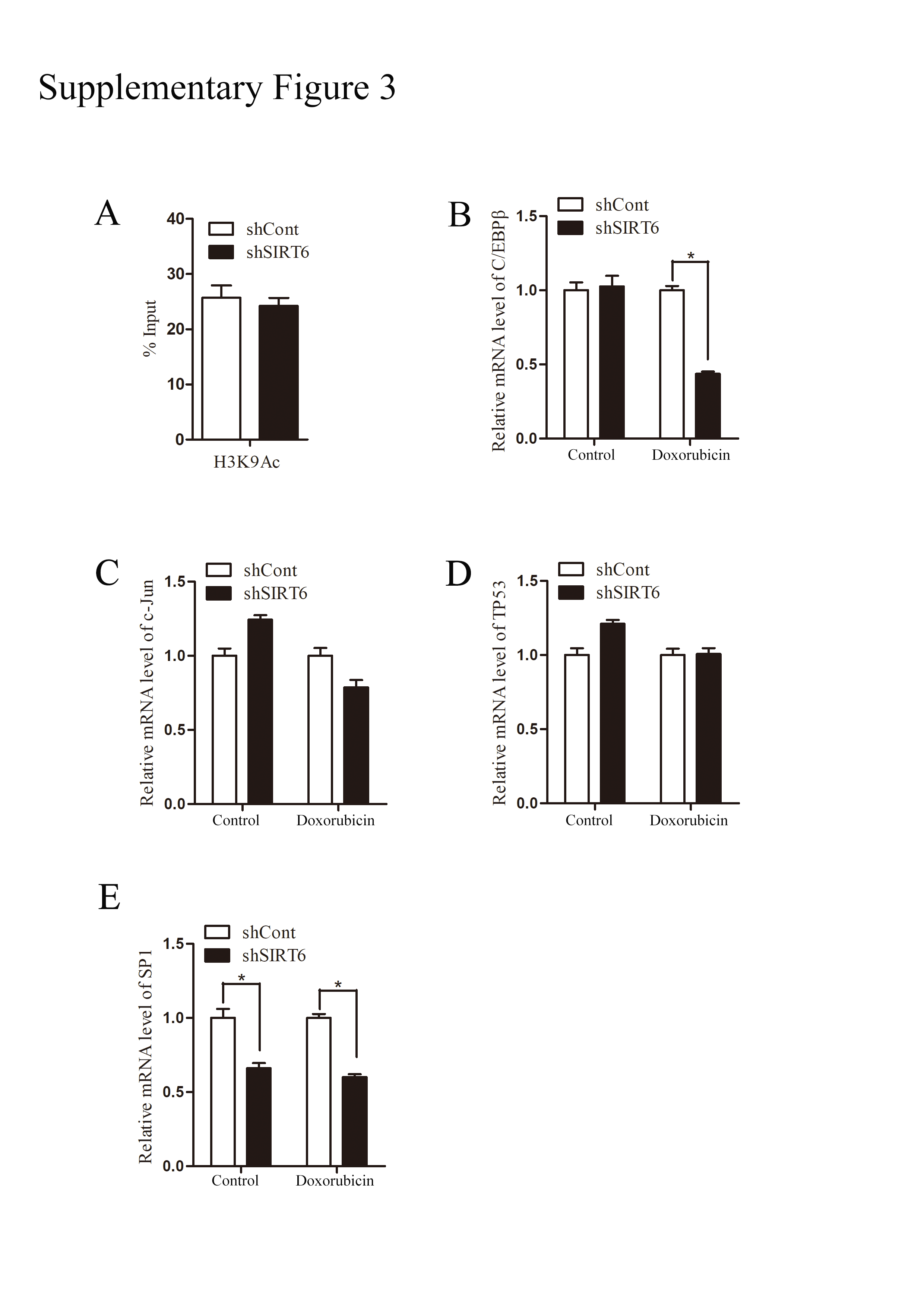

Supplement: Supplementary file 5 [file Image_3.TIF]
